# Editorial for the Special Issue “Oxidative Stress, Inflammation and Antioxidant Defense System in Psychiatric Disorders” in *Antioxidants* (2022–2023)

**DOI:** 10.3390/antiox13111307

**Published:** 2024-10-28

**Authors:** Pascal Steullet

**Affiliations:** Research Unit of Psychosis, Center of Psychiatric Neuroscience, Lausanne University Hospital, Prilly, 1008 Lausanne, Switzerland; pascal.steullet@chuv.ch

A growing body of evidence indicates that many genetic and environmental factors associated with psychiatric disorders affect redox homeostasis, mitochondria and energy metabolism, and neuroendocrine and immune systems in a complex synergistic manner, leading to oxidative stress and inflammation. Thus, signs of systemic oxidative stress, which have been reported in several psychiatric conditions, including schizophrenia, autism, bipolar and anxiety disorders, reflect homeostatic dysregulations. While oxidative stress is typically a consequence of these homeostatic dysregulations and the presence of systemic stressors, it has its own deleterious effects, altering redox-sensitive pathways and causing oxidative damage on lipids, proteins, and DNA. Therefore, redox dysregulations and oxidative stress may contribute to the emergence or progression of a disease and increase the risk of other comorbid disorders. Alterations in several different antioxidant systems are also commonly reported, possibly reflecting compromised antioxidant defenses and/or compensatory mechanisms in an attempt to mitigate oxidative stress. Antioxidant systems may also be dynamically altered along the successive stages of each disorder and influenced by various factors such as medication, age, sex, and lifestyle. Thus, it is incredibly challenging to interpret and/or identify the causes and consequences of systemic oxidative stress and alterations in antioxidant systems. Through systematic reviews, some of the contributions in this Special Edition aim to clarify the status of peripheral oxidative stress and antioxidants at different stages, according to different clinical characteristics of a disorder (e.g., schizophrenia, major depression disorder), as well as the impact of medication, age, and sex.

In the first contribution, the authors review published data (63 studies included) on peripheral oxidative stress and antioxidants in major depressive disorder (MDD), according to the disease stage and clinical features. The article highlights a considerably consistent increase in markers of oxidative damage in the blood of patients with MDD, which can be corrected via antidepressants and normalized in remitted patients. This suggests a link between oxidative stress and the severity of symptoms. By contrast, the reported alterations of non-enzymatic and enzymatic antioxidants vary across studies, reflecting heterogeneity among patients in terms of antioxidant defenses. The second contribution investigates the relationship between depressed mood and plasmatic levels of F2-Isoprostanes, a systemic marker of oxidative stress, in 568 aging adults (above 60 years old) from the general population. The authors find that a depressed mood and reduced self-reporting mental health are associated with high levels of F2-Isoprostanes in older individuals who are otherwise healthy. While no relationship between oxidative stress and mood is reported in a large cohort of younger adults of the general population [1], older adults show elevated oxidative stress levels with increased negative mood, similarly to clinical depression (contribution 1). The third contribution reviews the existing literature (76 studies included) on blood oxidative markers and antioxidants along the different stages of psychosis, from the early stages (ultra high-risk individuals and patients with early psychosis) to chronic schizophrenia. The current data indicate that individuals at risk of developing schizophrenia tend to have low antioxidant defenses, while patients with schizophrenia exhibit peripheral oxidative damage and reduced antioxidants, including reduced GSH peroxidase and superoxide dismutase activity. By contrast, a greater variability is found among patients with first psychotic episodes, although increased oxidative damage and reduced antioxidant defenses are often reported. In patients with schizophrenia, there is additionally an increased risk for atherosclerosis and cardiovascular disorder, which may be linked to pro-inflammatory and pro-oxidant states. A decrease in paraoxonase-1, an antioxidant HDL-associated protein that can hydrolyze oxidized LDL cholesterol, may account for a systemic pro-oxidative state and an increased risk for cardiovascular problems and atherosclerosis. The fourth contribution is a systematic review and meta-analysis (13 studies included) of the association between schizophrenia and blood paraoxonase and arylesterase activity, two enzymatic activities of circulating paraoxonase-1. The authors find overall no difference between patients and the controls for both paraoxonase and arylesterase activity. However, a subgroup analysis reveals significant reductions in arylesterase activity in studies on untreated and unmedicated patients. Moreover, the activities of paraoxonase and arylesterase are associated with age and LDL cholesterol, suggesting that a decrease in paraoxonase/arylesterase activity is present in subgroups of older patients with low levels of HDL cholesterol. Alterations in paraoxonase-1 may thus reflect a pro-oxidant state in specific subgroups of patients with schizophrenia at risk of cardiovascular disorder. The fifth contribution presents the hypothesis that the dysregulation of the apoptotic pathway could contribute to the pathophysiology of schizophrenia; it also proposes that some compounds known to mitigate apoptosis could be used as a potential therapeutical approach for schizophrenia. During neurodevelopment and brain maturation, some neurons undergo apoptosis-mediated death, and some synapses are eliminated by microglia via a mechanism known as synaptic pruning. There is some evidence for excessive synaptic pruning in schizophrenia, implicating the removal (by the microglia) of synapses tagged with molecules of the immune complement system. The mechanism of synaptic pruning is also linked to local apoptotic-like processes [2]. As there is evidence for the dysregulation of elements of the apoptosis pathway in schizophrenia, the authors suggest that apoptosis-like mechanisms at the level of synapses contribute to excessive pruning in schizophrenia. They also review a large variety of protecting molecules that could potentially prevent this mechanism. The sixth contribution focuses on attention-deficit/hyperactivity disorder (ADHD). Children with ADHD often have ophthalmologic abnormalities, which include functional vision problems [3]. The influence of medication for ADHD on retina functions and structure is, however, not clear. In this paper, the authors describe structural, functional, and neuronal alterations, together with microglial reactivity, astrogliosis, pro-inflammatory status, and blood–retinal barrier (BRB) hyperpermeability, in the retina of a rat model that displays ADHD-related behavioral anomalies. In this rat model, methylphenidate (MPH), a stimulant used to treat ADHD, reduces microgliosis, inflammatory status, and BRB hyperpermeability, but it does not recover the neuronal and functional anomalies of the retina relevant to ADHD. However, surprisingly, MPH impairs retinal function, neuronal cells and BRB integrity and promotes an inflammatory condition in the control rats. Thus, this study suggests that a misuse of MPH in subjects with no ADHD could be detrimental for retina function and integrity.

Together with the literature, the contributions of this Special Issue substantiate the idea that systemic oxidative stress is common among psychiatric disorders (see [4]), but we are still lacking cross-diagnosis comparisons to determine whether there exist some specific features/alterations for the different psychiatric conditions. The systematic reviews on schizophrenia and MDD (contributions 1 and 3) show high variability across studies and stages of the diseases regarding the levels of peripheral non-enzymatic and enzymatic antioxidants, which indicates that there is significant heterogeneity among patients in terms of both compromised and/or upregulated antioxidant systems in response to oxidative stress and the nature of the reactive oxygen species produced. Several contributions also emphasize the importance of age. Oxidative stress and antioxidant defense respectively increases and decrease with age, so the relationships between these peripheral parameters and disease and comorbid disorders may change in older individuals. The stratification of patients and healthy controls based on age is therefore strongly recommended. Likewise, it should be advised that data from males and females are analyzed separately due to significant sex differences in antioxidant capacities (e.g., GSH peroxidase [5,6]) and levels of oxidative stress (contribution 2). There is thus a need to move away from studies that examine oxidative damage and antioxidant capacities only at a diagnostic group level to studies that focus on classifying every individual based on multiple metrics associated with oxidative stress and antioxidants. Moreover, it is becoming clear that not every patient suffers from oxidative stress and/or responds similarly to stressors and homeostatic dysregulations.
